# History of the American Medical Association, from Its Organization up to January, 1855

**Published:** 1855-08

**Authors:** 


### BOOK NOTICES.

History of the American Medical Association, from its organization up to January, 1855. By N. S. DAVIS, M.D., Professor of Principles and Practice of Medicine and Clinical Medicine in Rush Medical College, etc. etc., to which is appended biographical notices, with portraits of the presidents of the Association and of the author. Philadelphia: Lippincott, Grambo & Co. 1855.

THE work of which the above is the title, appeared anonymously in the New Jersey Medical Reporter, where it attracted the notice of the profession to such an extent as to induce the publishers to present it to the public in its present form. The American Medical Association is certainly one of the most important organizations of the present day, and there is no one perhaps so intimately acquainted with its origin and the details of its history as Dr. Davis, and none better qualified to write its annals.

The object of the work is to awaken a deeper interest in the cause of medical progress, and we are quite confident that no one can read its pages without feeling a stronger attachment to the noble profession of medicine, and a more earnest desire to see its institutions sustained, its character elevated, and its honor advanced.

The portraits are excellent likenesses of men whom the profession have delighted to honor, and the biographical notices constitute an interesting portion of the work.

We believe there are a limited number of copies in the city, which can be obtained by addressing the author. H. A. J.

